# NOTCH1 activates the Wnt/β-catenin signaling pathway in colon cancer

**DOI:** 10.18632/oncotarget.19534

**Published:** 2017-07-25

**Authors:** Hideyuki Ishiguro, Tomotaka Okubo, Yoshiyuki Kuwabara, Masahiro Kimura, Akira Mitsui, Nobuyoshi Sugito, Ryo Ogawa, Takeyasu Katada, Tatsuya Tanaka, Midori Shiozaki, Koji Mizoguchi, Yosuke Samoto, Yoichi Matsuo, Hiroki Takahashi, Shuji Takiguchi

**Affiliations:** ^1^ Nagoya City University Graduate School of Medicine, Department of Gastroenterological Surgery, Mizuho-ku, Nagoya 467-8601, Japan

**Keywords:** colon cancer, β-catenin, NOTCH1, NICD1, prognosis

## Abstract

**Purpose and Methods:**

The translocation of β-catenin/CTNNB1 to the nucleus activates Wnt signaling and cell proliferation; however, the precise mechanism underlying this phenomenon remains unknown. Previous reports have provided evidence that NOTCH1 is involved in the Wnt signaling pathway. Therefore, we sought to determine the mechanism by which NOTCH1 influences the Wnt/β-catenin pathway. We constructed a vector expressing the NOTCH1 intracellular domain (NICD1) and transfected the vector into HCT116 which has low expression of NICD1. Furthermore, inhibition of NOTCH signal pathway in SW480 which has abundant NICD1 expression, was performed by transfection of siNICD1 or DAPT, gamma secretase inhibitor, treatment. In addition, we evaluated NICD1 and β-catenin localization in colon cancer cell lines and in 189 colon cancer tissue samples and analyzed the correlation between the nuclear localization of NICD1 and the clinicopathological features of colon cancer patients.

**Results:**

Immunohistochemical assays demonstrated that NICD1 and β-catenin exhibited a similar localization pattern in colon cancer tissues. In addition, we found that NICD1 induced the translocation of β-catenin to the nucleus and that NICD1 and β-catenin co-localized in the nucleus. Overexpression of NICD1 increased luciferase activity of Wnt signal pathway. On the other hand, reduction of NICD1 reduced luciferase activity of Wnt signaling pathway. In the 189 analyzed colon cancer cases, multivariate COX regression analysis demonstrated the independent prognostic impact of nuclear localization of NICD1(p=0.0376).

**Conclusion:**

NOTCH1 plays a key role in the Wnt pathway and activation of NOTCH1 is associated with the translocation of β-catenin to the nucleus.

## INTRODUCTION

Recent progress in cancer research has revealed that β-catenin/CTNNB1, which functions in cell-to-cell adhesion and Wnt signaling, is a key contributor to carcinogenesis in various tissues, including the colon, liver, ovary, and skin [1–6]. Cellular β-catenin is normally degraded by complexes composed of glycogen synthase kinase-3β (GSK-3β), Axin, and adenomatous polyposis coli (APC) [5, 7–9]. Mutations in APC, Axin, or β-catenin promote the accumulation of β-catenin and the formation of complexes composed of β-catenin and Tcf/Lef [10–13]. The β-catenin and Tcf/Lef complex translocates to the nucleus where it transactivates downstream genes [5, 10, 11] that promote the transformation of a normal cell into a tumor cell. Although several genes targeted by this complex, including c-myc and cyclin D1, have been identified, the molecular mechanism underlying β-catenin-Tcf/Lef signaling has yet to be fully characterized [14–16]. Notably, the mechanism mediating the critical event of β-catenin/CTNNB1 translocation to the nucleus remains unclear.

The NOTCH signaling pathway plays a critical role in tissue development and homeostasis by regulating cell fate, proliferation, differentiation, and apoptosis [17, 18]. NOTCH1 has been reported to act as a transcriptional activator that plays essential roles in the development of multiple types of cancers [17, 19–21]. The NOTCH family includes 4 receptors, NOTCH1-4, whose ligands include JAG1, JAG2, DLL1, DLL3, and DLL4. All of the NOTCH receptors have an extracellular domain containing multiple epidermal growth factor-like repeats and an intracellular region composed of a RAM domain, ankyrin repeats, and a C-terminal PEST domain [22]. NOTCH receptors and their ligands have been shown to be up-regulated in cervical, lung, colon, renal, and pancreatic cancers as well as in acute myeloid leukemia and Hodgkin and large-cell lymphomas [17, 19–21].

In this study, we evaluated the involvement of NOTCH1 in the Wnt/CTNNB1 pathway using a vector expressing the NOTCH1 intracellular domain (NICD1). Several reports have provided evidence suggesting that NOTCH1 functions as a negative regulator of the Wnt signaling pathway [23]. However, there are also recent reports suggesting that NOTCH1 is overexpressed in patients with colon cancer [24–26] and that a reduction in NOTCH1 expression induces apoptosis in pancreatic cancer cells [21].

Studies investigating whether NOTCH1 negatively or positively regulates the Wnt signaling pathway have presented conflicting results. Here, we demonstrated that NOTCH1 acts as an oncogene in colon cancer by activating Wnt signaling.

## RESULTS

### NOTCH1 and β-catenin exhibited a similar localization pattern in colon cancer cells

**Figure 1:**
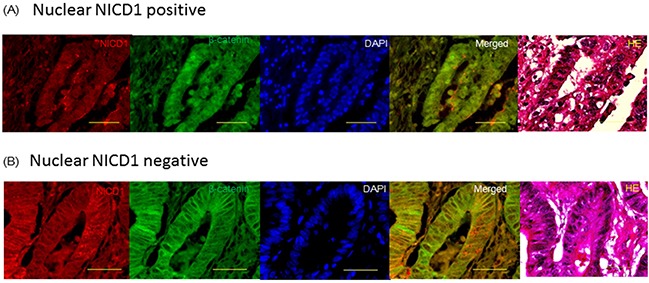
Confocal imaging of immunohistochemistry analysis of β-catenin and NICD1 in colon cancer tissues Representative image of β-catenin and NICD1 localization in colon cancer tissue samples. DAPI, Merged image of NICD1 and β-catenin and HE stain. The antibody used (ab8925, from Abcam) actually recognizes the active form of Notch1 receptor, exposed after cleavage by γ-secretase. Yellow bar indicates 50um. **(A)** Nuclear NICD1 positive pattern: NICD1 and β-catenin were expressed in the nucleus of colon cancer cell. Merged image indicated that NICD1 and β-catenin were co-expressed in the same colon cancer cell. **(B)** Nuclear NICD1 negative pattern: NOTCH1 and β-catenin were expressed in the cytoplasm of colon cancer cell. Merged image indicated that NOTCH1 and β-catenin were co expressed in the same colon cancer cell.

First, we examined the distribution of β-catenin and NOTCH1 in colon cancer tissue samples using co-immunohistochemistry. Interestingly, the distribution of the 2 proteins was similar in colon cancer cells (Figures 1A and 1B). In the majority of the colon cancer tissue samples, NOTCH1 and β-catenin were co-localized.

### Association between NOTCH1 localization and clinicopathological parameters

We examined the localization of NOTCH1 in 189 colon cancer tissue samples using immunohistochemistry. In approximately 50% of the colon cancer samples, NOTCH1 was strongly expressed in the nucleus (Figure 1A). Intracellular domain of NOTCH1 is called NICD1 that localizes in the cytoplasm or nucleus of the cells.

In addition, we observed a statistically significant association between nuclear NICD1 expression and TNM staging (Table 1). Nuclear NICD1 expression in colon cancer tissue samples was associated with a significantly greater prevalence of stage T3-4 disease compared with stage T1-2 disease (p=0.0013) (Table 1).

**Table 1:** Correlation of NICD1 IHC in colon cancer with clinicopathological factors, including patient and tumor characteristics

Characteristics	No. of patients(n=189) Nuclear NICD1(+)	P-value
**Tumor status**		
T1	6/10	
T2	12/27	
T3	107/141	
T4	8/11	
T12 vs T34	18/37 vs 115/152	0.0013
**Lymph node status**		
N(-)	65/93	
N(+)	68/96	0.8874
**Lymphatic invasion**		
Negative	14/21	
Positive	119/168	0.6934
**Blood vessel invasion**		
Negative	36/48	
Positive	97/141	0.4161

### Nuclear NICD1 expression correlates with poor prognoses in colon cancer patients

Next, we examined whether NICD1 nuclear localization was associated with patient survival after surgery. Kaplan-Meier survival curves demonstrated that the survival rate was significantly lower in patients with nuclear NICD1 expression (p=0.0027 by log-rank test) (data not shown). The prognostic value of various clinicopathological factors was evaluated using univariate Cox regression analysis (Table 2). In addition to the correlation of NICD1 nuclear localization with the T factor (p=0.0237) and N factor (p=0.0167) in the TNM staging system, nuclear NICD1 expression was significantly associated with a poor prognosis (p=0.0217). In contrast, lymphatic invasion and venous invasion were not significantly associated with survival (p=0.2235 and p=0.6507, respectively) (Table 2).

**Table 2:** Univariate and multivariate Cox regression analysis of clinicopathologic factors and nuclear NICD1 in colon cancer

Variables	HR	(95% CI)	Unfavorable/favorable	*P*-value
**Univariate Cox regression analysis**				
Primary tumor(T factor)	3.355	1.175-9.523	T4/T1-3	0.0237
Lymph Node metastasis(N factor)	2.421	1.173-5.000	N(+)/N0	0.0167
Ly factor	3.448	0.470-25.000	Positive/Negative	0.2235
V factor	1.212	0.526-2.793	Positive/Negative	0.6507
Nuclear NICD1	2.344	1.133-4.850	Positive/Negative	0.0217
**Multivariate Cox regression analysis**				
Primary tumor(T factor)	4.115	1.432-11.90	T4/T1-3	0.0086
Lymph Node metastasis(N factor)	2.293	1.100-4.784	N(+)/N0	0.0267
Nuclear NICD1	2.181	1.046-4.549	Positive/Negative	0.0376

Abbreviation: HR, hazard ratio; 95%CI, 95%confidence interval

The associations of the T factor, N factor, lymphatic and venous invasion, and nuclear expression of NICD1 with prognosis and survival were further analyzed using Cox proportional hazards modeling. Nuclear NICD1 localization and the T factor (p=0.0086) were identified as significant and independent prognostic indicators (p=0.0376) of survival in postoperative colon cancer patients.

The hazard ratio for reduced survival associated with positive nuclear NICD1 expression compared with negative nuclear NICD1 expression was 2.181 (95% confidence interval: 1.046-4.549) (Table 2). These findings strongly suggest that nuclear NICD1 expression have an impact on the survival of postoperative colon cancer patients.

### NICD1 induced β-catenin translocation to the nucleus and cell proliferation in colon cancer cells

**Figure 2:**
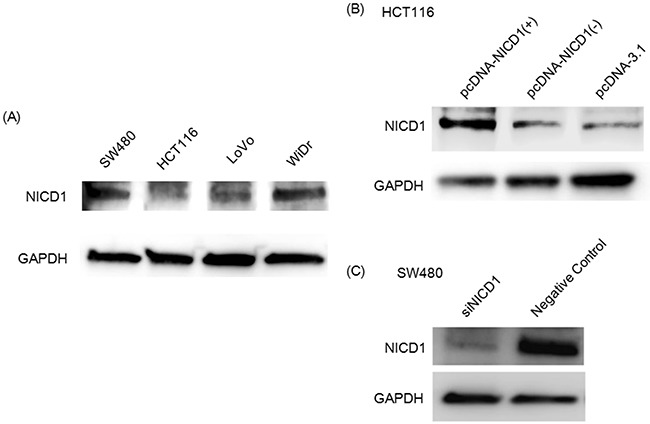
**(A)** Wetern blot analysis of NICD1 expression in 4 colon cancer cell lines. The NICD1 band is approximately 120 kD. GAPDH was used as internal control. **(B)** Western blot analysis of HCT116 transfected with the pcDNA3.1-NICD1(+), pcDNA3.1-NICD1(-)(Mock) or pcDNA3.1 vector. **(C)** Western blotting analysis of NICD1 expression in siNICD1-transfected cells. NICD1 expression levels were suppressed in cells transfected with siNICD1 compared with Negative Control.

To determine the cell line to transfect NICD1 expression vector, we examined the status of NICD1 expression in the four colon cancer cell lines (Figure 2A). In HCT116, NICD1 expression was lower than other cell lines. On the other hand, in SW480, NICD1 expression was abundant among four colon cancer cell lines. Next, to evaluate the effect of NICD1 expression in colon cancer cells, we constructed an NICD1 expression vector. We confirmed the expression of the NICD1 expression vector (pcDNA3.1-NICD1) in the HCT116 colon cancer cell line using western blot analysis (Figure 2B). And we inhibited NICD1 expression in SW480 colon cancer cells using siRNA. Western blotting using extracted whole cell lysate showed that NICD1 levels were decreased in siNICD1-transfected SW480 cells compared with cells transfected with the control siRNA (Figure 2C).

**Figure 3:**
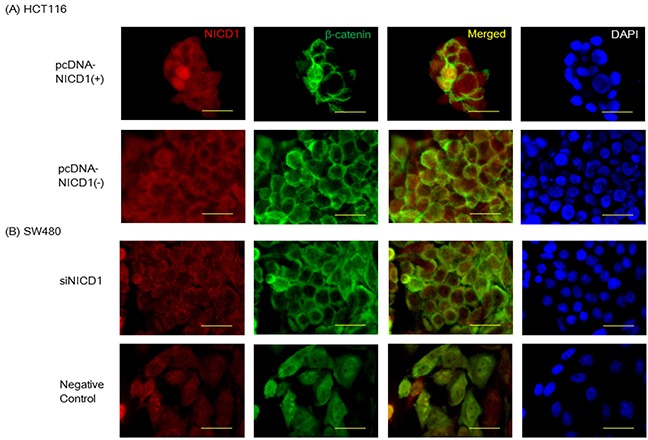
**(A)** Confocal imaging of NICD1 and β-catenin immunohistochemistry staining in HCT116 cells transfected with pcDNA3. 1NICD1(+) or pcDNA3.1-NICD1(-). In HCT116 cells transfected pcDNA3.1NICD1(+), β-catenin was robustly expressed. **(B)** Confocal imaging of NICD1 and β-catenin immunohistochemistry staining in SW480 cells transfected with siNICD1 or Negative Control. In SW480 cells transfected siNICD1, β-catenin expression in the nucleus was reduced. Yellow bar indicates 30um.

Furthermore, we analyzed HCT116 and SW480 colon cancer cells using immunohistochemical assays with antibodies against NICD1 and β-catenin. NICD1 localized to the cell membrane and cytoplasm of HCT116 (Figure 3A) transfected with control vector. Similarly, β-catenin/CTNNB1 was strongly expressed in the cytoplasm and at the cell membrane in HCT116 cell lines (Figure 3A). In addition, we observed low levels of endogenous NICD1 expression in the nucleus of HCT116 (Figure 3A). To further analyze the effect of NICD1 expression, we compared the localization patterns of β-catenin in HCT116 cells transfected with the pcDNA3.1-NICD1(+) or pcDNA-Mock(-) (as a control) vector (Figure 3A). Nuclear β-catenin levels were increased in NICD1-transfected HCT116 cells compared with cells transfected with the control plasmid (Figure 3A).

On the other hand, endogenous NICD1 localized to the nucleus and cytoplasm of SW480 transfected with control siRNA (Figure 3B). Similarly, β-catenin/CTNNB1 was strongly expressed in the cytoplasm and at the nucleus in SW480 cell lines (Figure 3B). To determine the effect of siNICD1 expression, we compared the localization patterns of β-catenin in SW480 cells transfected with the siNICD1 or negative control siRNA (as a control) (Figure 3B). Nuclear β-catenin levels were decreased in siNICD1-transfected SW480 cells compared with cells transfected with the control siRNA (Figure 3B). In summary, nuclear β-catenin were observed in NICD1-expressing colon cancer cells, but not in cells transfected with the control vector (Figure 3A). Reduction of nuclear NICD1 decreased nuclear β-catenin in the colon cancer cells.

### NICD1 controlled Wnt signal in colon cancer cells

**Figure 4:**
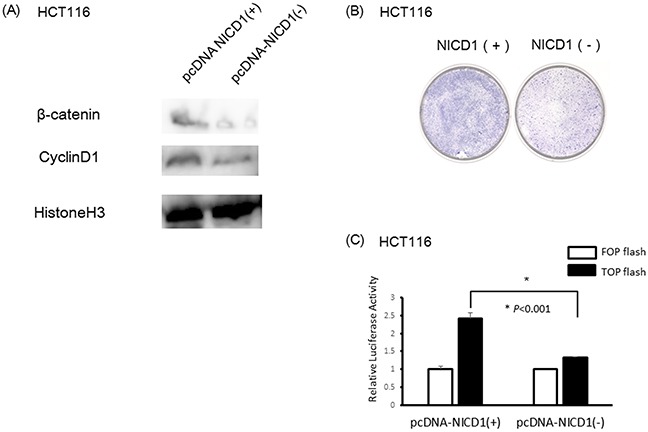
Induction of NICD1 into HCT cells with lower endogenous NICD1 **(A)** Western blotting analysis of NICD1, β-catenin and Cyclin D1 expression in pcDNA3.1-NICD1(+) or pcDNA3.1-NICD1(-)(Mock) -transfected cells. β-catenin and Cyclin D1 expression levels in the nucleus were increased in cells transfected with pcDNA3.1-NICD1(+). **(B)** Colony formation assay using HCT116 cells transfected with pcDNA3.1-NICD1(+) or pcDNA3.1-NICD1(-) (control). Colony formation in HCT116 cells transfected with pcDNA3.1-NICD1 was enhanced compared with HCT116 cells transfected with the control vector. **(C)** Relative β-catenin / TCF luciferase activity of HCT116 cells transfected with pcDNA3.1-NICD1(+) or pcDNA3.1-NICD1(-) (control).

To determine the effect of NICD1 on genes activated downstream of Wnt signaling, we induced NICD1 expression in HCT116 colon cancer cells using NICD1-expressing vector. The results of Western blotting using extracted nuclear protein revealed that nuclear β-catenin levels were increased in pcDNA NICD1(+)-transfected HCT116 cells compared with cells transfected with the control vector (Figure 4A). Furthermore, CyclinD1, a downstream target of the Wnt signaling pathway, increased in NICD1-induced HCT116 cells (Figure 4A).

Giemsa staining demonstrated that cell proliferation was enhanced in HCT116 colon cancer cells compared with the control cells (Figure 4B). Moreover, TCF/β-catenin activity increased in HCT116 cells transfected pcDNA NICD1(+) compared with control vector using luciferase assay (Figure 4C).

**Figure 5:**
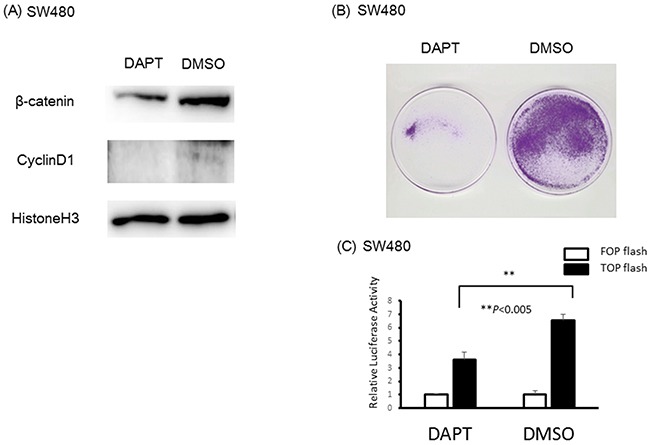
Reduction of NICD1 into SW480 cells with abundant endogenous NICD1 **(A)** β-catenin and Cyclin D1 expression in the nucleus of SW480 cells treated with DAPT or DMSO(control). β-catenin and Cyclin D1 expression levels in the nucleus of SW480 cells were suppressed in cells transfected with DAPT. **(B)** Colony formation assay using SW480 cells treated with DAPT or DMSO (control). Colony formation in SW480 cells treated with DAPT was suppressed compared with SW480 cells treated with DMSO (control). **(C)** Relative β-catenin / TCF luciferase activity of SW480 cells treated with DAPT or DMSO (control).

Next, to inhibit the translocation of NICD1 to the nucleus in SW480 colon cancer cells with abundant nuclear NICD1, we used DAPT which is a gamma-secretase inhibitor. DAPT inhibits the cutting of the site between NICD1 and NECD (NOTCH1 extracellular domain). The results of Western blotting using extracted nuclear protein revealed that nuclear β-catenin and Cyclin D1 levels were decreased in DAPT-treated SW480 cells compared with cells transfected with DMSO as a control (Figure 5A). Cell proliferation was reduced in SW480 colon cancer cells compared with the control cells (Figure 5B). TCF/β-catenin activity decreased in SW480 cells treated DAPT compared with control using luciferase assay (Figure 5C).

## DISCUSSION

NOTCH1 activation is mediated by gamma secretase-mediated cleavage of the NOTCH S3 site [31]. S3 cleavage releases the NOTCH1 intracellular domain (NICD1) from the membrane, and NICD1 subsequently translocates to the nucleus, where it functions as a transcriptional activator [32, 33]. Until recently, it was unclear whether NOTCH1 functions as an oncogene or a tumor suppressor in cancer. Nicolas et al. reported that NOTCH1 acts as a tumor suppressor in mammalian skin [34], and other groups have reported that NOTCH1 acts as a tumor suppressor in esophageal cancer [35] and hepatocellular carcinoma [36]. In contrast to these findings, there have been recent reports that NOTCH1 functions as an oncogene in melanoma [37], breast cancer [38, 39], pancreatic cancer [21], and lymphoma [40].

The expression of NOTCH1 has been reported to be up-regulated in colon cancer tissue [25, 26]. Furthermore, NOTCH1 knockdown significantly inhibits proliferation and colony formation in colon cancer cell lines [25]. These findings suggest that activation of the NOTCH signaling pathway plays a role in colon cancer and that NOTCH1 possesses oncogenic activity. Our data revealed that translocation of NICD1 into the nucleus was correlated with a poor prognosis in colon cancer patients (Table 2). In addition, colony formation assays demonstrated that translocation of NICD1 into the nucleus enhanced cell proliferation in colon cancer cells. Consistent with these findings, other proteins involved in NOTCH signaling, including JAG1-2, HES1, DLL1 and DLL3, are activated in colon adenocarcinoma [24, 41–43]. Together, these findings suggest that NOTCH signaling is activated in colon cancer. Figure 2A revealed that most colon cancer cell lines expressed NICD1. We used SW480 with especially abundant NICD1 expression and HCT116 with lower expression of NICD1 compared with other colon cancer cell lines.

Further investigation into the role of NOTCH1 in colon cancer might help to elucidate its role in the Wnt signaling pathway.

There are many reports describing the crosstalk between the Wnt/β-catenin and NOTCH signaling pathways [23, 35, 44–47]. Nevertheless, the role of NOTCH1 in Wnt signaling remains controversial. One hypothesis is that NOTCH interacts with β-catenin at the cell membrane in stem and colon cancer cells and inhibits the accumulation of β-catenin [23]. However, β-catenin accumulation in the nucleus and cytoplasm is frequently observed in colon cancer cases with APC mutations [48, 49]. These findings suggested discrepancies related to the hypothesis that NOTCH1 and β-catenin localize to the nucleus in colon cancer cells. Our data revealed that NOTCH1 and β-catenin co-localize in the colon cancer cells. (Figure 1A and 1B)

Membrane-bound NOTCH1 binds to and inhibits unphosphorylated (active) β-catenin [23]. However, once NOTCH1 (NICD1) is activated by gamma-secretase, β-catenin transcriptional activity might be activated by interacting with the activated NICD1.

Transfecting colon cancer cells with the NICD1 expression vector promoted the translocation of β-catenin into the nucleus and induced cell proliferation. As β-catenin lacks a nuclear localization signal (NLS), it might translocate to the nucleus by interacting with NICD1, which contains an NLS. In fact, luciferase assay using TOPFLASH indicated that NICD1 induced TCF/β-catenin activity. Thus, we hypothesize that NICD1 promotes Wnt signaling by mediating the translocation of β-catenin to the nucleus and that NICD1 functions as an oncogene in colon cancer.

Interestingly, the accumulation of NICD1 in the nucleus induces the transformation of rat kidney cells (RKE cells) by up-regulating cyclin D1 expression [50]. Several genes, including cyclin D1 and c-myc, are known to be up-regulated by the activation of β-catenin [14–16]. As cyclin D1, in conjunction with retinoblastoma protein, is involved in cell cycle regulation, the up-regulation of cyclin D1 expression might promote uncontrolled cell proliferation [15, 16]. Moreover, there are some reports indicating that c-Myc is a transcriptional target of NOTCH1 [51–53].

Overexpression of components of the NOTCH signaling pathway is often associated with poor outcomes or tumor metastasis [54]. Together, these findings indicate that NOTCH signaling is oncogenic in a variety of human tumors. Consistent with these findings, our data suggest that NOTCH1 exerts oncogenic activity in colon cancer, as the nuclear translocation of NOTCH1 correlated with the T factor in TNM staging and with a poor prognosis.

Our data indicate that the activation of the NOTCH pathway promotes colon cancer. Therefore, we hypothesize that NOTCH1 acts as an oncogene in colon cancer.

NOTCH1 mutations have been observed in various cancers [55, 56]. The observation that β-catenin and NOTCH1 do not always co-localize in immunohistochemical assays might be attributed to mutations in NOTCH1 itself. However, to the best of our knowledge, there have been no reports of NOTCH1 mutations in colon cancer.

The association between NOTCH1 and colon cancer was confirmed by the results of experiments using SW480 human colon cancer cells, which express high levels of β-catenin. siRNA-mediated NICD1 depletion in SW480 cells reduced Wnt signaling activity mediated by the β-catenin-Tcf/Lef complex, thereby significantly reducing cyclin D1 expression. Together, the results of our experiments using the NICD1 expression vector strongly suggest that NOTCH1 plays an important role in the β-catenin signaling pathway.

Upon the activation of gamma secretase, NOTCH1 is cleaved at its S3 site, resulting in the release of activated NICD1. In our experiments, activated NOTCH1 (the cleaved NICD1) interacted with β-catenin and translocated to the nucleus. Moreover, cell proliferation was induced in pcDNA3.1 NICD1-transfected HCT116 colorectal cancer cells expressing low levels of β-catenin. However, additional molecular functions of NOTCH1 in colon cancer have yet to be characterized. We subsequently investigated the association of NOTCH1 expression in resected tumor tissues samples with the clinicopathological features and prognosis of colon cancer patients.

Our findings indicate that the role of NOTCH1 in colon cancer progression merits further investigation. Although the precise molecular mechanisms underlying the up-regulation of NOTCH1 expression and the activation of NICD1 have yet to be elucidated, our data suggest that NOTCH1 is a candidate prognostic molecular marker and a promising molecular target for the development of effective therapeutic options for patients with colon cancer.

## MATERIALS AND METHODS

### Cells and reagents

The HCT116, SW480, LoVo and WiDr human colon cancer cell lines were obtained from American Type Culture Collection (ATCC, Rockville, MD). SW480 cells, LoVo, WiDr and HCT116 cells were cultured in L15, Ham F12, EMEM and RPMI medium, respectively, supplemented with 5-10% fetal bovine serum and a 1% antibiotic/antimycotic solution (Sigma Chemical Co., St. Louis, MO) at 37°C in a humidified atmosphere. An antibody ab8925 (Abcam, Cambridge, UK) which actually recognizes the active form of Notch1 receptor, exposed after cleavage by γ-secretase in the methods, was obtained as previously described [27].

Anti-β-catenin was purchased from BD Transduction Laboratories (Japan) and anti-GAPDH was purchased from Cell Signaling Technology, Inc. (Dancers, MA).

### Patients and tumor samples

Colorectal cancer tissue samples were obtained from 189 patients who had undergone surgery at the Nagoya City University Hospital (Nagoya, Japan) between January 1998 and December 2007. The resected specimens were staged by pathological evaluation according to the UICC guidelines for clinical and pathological studies of colon cancer (Table 1). Colon cancer was confirmed in all of the tumor tissue samples by the Clinicopathology Department. The samples were used after obtaining written consent from the patients.

### Protein isolation and immunoblot analysis

Total cell lysates were prepared in 2% SDS lysis buffer with 330 mM Tris-HCl (pH 8.8), 2% SDS, 10% glycerol, and 1 Mini Protease Inhibitor Cocktail Tablet (Roche Diagnostics Corp., Tokyo, Japan). Cytoplasmic and nuclear extracts were prepared using NE-PER Nuclear and Cytoplasmic Extraction Reagents (Thermo Fisher scientific Inc., Waltham, MA, USA) according to the manufacturer's instructions. Equal amounts of protein were separated by electrophoresis on 10% Tris-glycine gels. Western blot analyses were performed as previously described [28], and the immunoreactive protein bands were visualized using a chemiluminescence detection reagent. To harvest the cells for immunocytochemistry staining, cytospin slides were prepared with a Cytospin cytology centrifuge. NOTCH1 and β-catenin were detected as previously described [27, 29].

### Plasmids and transient transfection

The NICD1 cDNA expression vector pcDNA3.1-NICD1 was constructed to express the region of NOTCH1 between the codon encoding Ser-1748 and the stop codon. To examine the effects of NICD1 on Wnt transcriptional activity, NICD1-expressing cells were plated in 6-well plates and transiently transfected with 3 μg of the pcDNA3.1-NICD1 plasmid with 9 μL of FuGENE 6 (Promega Corporation, Madison, WI, USA) according to the manufacturer's protocol. The cells were harvested 72 h after transfection, and the localization of NICD1 was analyzed using immunohistochemistry and western blot assays.

### SiRNA transfection

For inhibition of Notch1 in HCT116 cells and SW480, Notch1-siRNA and negative control-siRNA and Lipofectamine® RNAiMAX transfection Reagent were purchased from Thermo Fisher Scientific Inc. (Waltham, MA, USA). Cells were plated in 10cm or 6cm dish and transiently transfected with Lipofectamine® RNAiMAX transfection Reagent according to the manufacturer's protocol. The cells were incubated with transfection mixtures containing 20 nM of Notch1-siRNA or negative control-siRNA for 72 hours.

### DAPT treatment

Cells were treated with 25 μmol/L DAPT (D5642: Sigma-Aldrich, USA) and 0.1% dimethyl sulfoxide (DMSO) (Sigma, St. Louis, MO, United States) as a control. After treatment for 72 h, all cells were collected for protein extraction.

### Immunocytochemistry analysis

We transfected HCT116 and SW480 cells with the pcDNA3.1-NICD1 expression vector. The transiently transfected cell lines were plated on chamber slides and fixed with 4% paraformaldehyde in PBS. The cells were subsequently permeabilized using 0.1% Triton X-100 in PBS for 3 min at 4°C and incubated with blocking solution (2% BSA in PBS) for 30 min at room temperature to block nonspecific antibody-binding sites. Then, the cells were incubated with a mouse antibody against NOTCH1 (diluted 1:100 in blocking solution). The primary antibodies were detected using a goat anti-rabbit secondary antibody conjugated to rhodamine, and the stained cells were imaged using a laser scanning confocal imaging system, BZX-700(KEYENCE Corp., Osaka Japan). We confirmed that the transfected cells expressed the NICD protein using western blotting as previously described [30].

### Western blot analysis

Equal amounts of total cell lysate solubilized in Laemmli's sample buffer were separated using SDS-PAGE and transferred to Immobilon-P filters (Millipore Corp., Bedford, MA). The filters were incubated with anti-NOTCH1 and subsequently incubated with horseradish peroxidase-conjugated secondary antibodies [anti-mouse IgG and anti-rabbit IgG (Cell Signaling Technology, Beverly, MA) and anti-goat IgG (MBL, Nagoya, Japan)]. The reactions were visualized using an enhanced chemiluminescence system, Amersham Imager 600 (GE Healthecare UK Ltd. Buckinghamshire, UK).

### Immunohistochemistry

Colon cancer sections were obtained from Nagoya City University Hospital (Nagoya, Japan). For antigen retrieval, deparaffinized sections were boiled in citrate buffer (10 mM sodium citrate buffer, pH 6.0) prior to incubation with the primary antibodies. NOTCH1 and β-catenin protein levels were examined using rabbit polyclonal antibodies (ab8925) and mouse monoclonal antibodies (Abcam, Cambridge, UK). Nuclear CyclinD1 and HistonH3 protein levels were examined using rabbit monoclonal antibodies (Cell Signaling Technology, Inc. Dancers, MA, USA)

Antibody staining was conducted using the peroxidase-based DAKO EnVision System.

### Colony formation assay

Cell proliferation was analyzed in the HCT116 and SW480 cancer cell lines using colony formation assays. The cells were plated in 10 cm dishes (2×10^6^ cells/dish) for 24 h and subsequently transfected with pcDNA3.1-NICD1 or pcDNA3.1-Mock plasmid using 10 μg of plasmid DNA and 30ul of FuGENE6® Transfection Reagent (Promega Corporation, Madison, WI, USA). After the cells were transfected for 24 h, they were diluted 1:8 and cultured for 14 days in the presence of 800 μg/ml geneticin (G418).

### Immunofluorescence

HCT116 and SW480 cells were grown on Lab-Tek chamber slides (Nalge Nunc International K.K., Rochester, NY). The cells were washed once with phosphate-buffered saline, fixed with 4% paraformaldehyde for 15 min, and permeabilized using 0.2% Triton X-100 on ice for 5 min. Cells were then incubated with blocking solution (3% BSA in PBS) for 60 min at room temperature. The cells were subsequently incubated for overnight at 4°C with rabbit anti-NOTCH1 (ab8925) (1:400 dilution) in Tris-buffered saline with 3% bovine serum albumin. Goat anti-rabbit Cy3® (IgG H&L)-preadsorbed ab6939 (Abcam) was visualized in the red channel, and goat anti-mouse Alexa Fluor® 488 (IgG H&L) (Abcam) was visualized in the green channel. Microscopy analysis and image acquisition were conducted using a laser scanning confocal imaging system, BZX-700(KEYENCE Corp., Osaka Japan).

### Indirect immunofluorescence staining and confocal laser microscopy

Cells were seeded onto 4-chamber slides before being fixed and permeabilized. The fixed cells were incubated with anti-NOTCH1and anti-β-catenin (all diluted 1:100) for 1 h at room temperature. The cells were subsequently incubated with the Cy3-conjugated goat anti-rat and FITC-conjugated goat anti-rabbit secondary antibodies diluted 1:100 for 30 min at room temperature. The cell nuclei were stained with ProLong Gold antifade reagent with 4′,6-diamidino-2-phenylindole (DAPI) (Invitrogen). Protein expression and localization were examined using a confocal microscopy system (FluoView FV500, Olympus).

### TOP FLASH/FOP-FLASH reporter assay

TOP-Flash reporter and pTK-RL plasmids were transiently co-transfected into colon cancer cells (5×10^4^) in 24well plates, and the activities of both firefly and Renilla luciferase reporters was determined at 72 hours after transfection using a Dual Luciferase Assay Kit (Promega, Madison, WI, USA) according to the manufacturer's instructions. The TOP-FLASH reporter activity is presented as the relative ratio of firefly luciferase activity to Renilla luciferase activity. All experiments were performed three times in triplicate.

### Statistical analysis

The biostatistical analyses were conducted using Stat-View software (Abacus Concepts, Berkeley, CA). Student's t-test was employed to determine the optimal cut-off value for comparing gene expression levels between 2 groups. The associations between various clinicopathological characteristics and the localization of NOTCH1 and β-catenin were evaluated using Fisher's exact test. Kaplan-Meier estimates of overall survival were compared via the log-rank test. Cox regression analysis of potential prognostic indicators of survival was used to identify independent factors that significantly affect survival. All tests were two-tailed, and p<0.05 was considered statistically significant.

## References

[1] Nusse R, Varmus HE (1992). Wnt genes. Cell.

[2] van den Heuvel M, Nusse R, Johnston P, Lawrence PA (1989). Distribution of the wingless gene product in Drosophila embryos: a protein involved in cell-cell communication. Cell.

[3] Wieschaus E, Riggleman R (1987). Autonomous requirements for the segment polarity gene armadillo during Drosophila embryogenesis. Cell.

[4] Funayama N, Fagotto F, McCrea P, Gumbiner BM (1995). Embryonic axis induction by the armadillo repeat domain of beta-catenin: evidence for intracellular signaling. J Cell Biol.

[5] Nakamura Y (1997). Cleaning up on beta-catenin. Nat Med.

[6] Rubinfeld B, Robbins P, El-Gamil M, Albert I, Porfiri E, Polakis P (1997). Stabilization of beta-catenin by genetic defects in melanoma cell lines. Science.

[7] Behrens J, Jerchow BA, Wurtele M, Grimm J, Asbrand C, Wirtz R, Kuhl M, Wedlich D, Birchmeier W (1998). Functional interaction of an axin homolog, conductin, with beta-catenin, APC, and GSK3beta. Science.

[8] Hamada F, Tomoyasu Y, Takatsu Y, Nakamura M, Nagai S, Suzuki A, Fujita F, Shibuya H, Toyoshima K, Ueno N, Akiyama T (1999). Negative regulation of Wingless signaling by D-axin, a Drosophila homolog of axin. Science.

[9] Hart M, Concordet JP, Lassot I, Albert I, del los Santos R, Durand H, Perret C, Rubinfeld B, Margottin F, Benarous R, Polakis P (1999). The F-box protein beta-TrCP associates with phosphorylated beta-catenin and regulates its activity in the cell. Curr Biol.

[10] Korinek V, Barker N, Morin PJ, van Wichen D, de Weger R, Kinzler KW, Vogelstein B, Clevers H (1997). Constitutive transcriptional activation by a beta-catenin-Tcf complex in APC−/− colon carcinoma. Science.

[11] Morin PJ, Sparks AB, Korinek V, Barker N, Clevers H, Vogelstein B, Kinzler KW (1997). Activation of beta-catenin-Tcf signaling in colon cancer by mutations in beta-catenin or APC. Science.

[12] Nakamura T, Hamada F, Ishidate T, Anai K, Kawahara K, Toyoshima K, Akiyama T (1998). Axin, an inhibitor of the Wnt signalling pathway, interacts with beta-catenin, GSK-3beta and APC and reduces the beta-catenin level. Genes Cells.

[13] Rubinfeld B, Albert I, Porfiri E, Munemitsu S, Polakis P (1997). Loss of beta-catenin regulation by the APC tumor suppressor protein correlates with loss of structure due to common somatic mutations of the gene. Cancer Res.

[14] He TC, Sparks AB, Rago C, Hermeking H, Zawel L, da Costa LT, Morin PJ, Vogelstein B, Kinzler KW (1998). Identification of c-MYC as a target of the APC pathway. Science.

[15] Shtutman M, Zhurinsky J, Simcha I, Albanese C, D'Amico M, Pestell R, Ben-Ze'ev A (1999). The cyclin D1 gene is a target of the beta-catenin/LEF-1 pathway. Proc Natl Acad Sci U S A.

[16] Tetsu O, McCormick F (1999). Beta-catenin regulates expression of cyclin D1 in colon carcinoma cells. Nature.

[17] Miele L (2006). Notch signaling. Clin Cancer Res.

[18] Bray SJ (2006). Notch signalling: a simple pathway becomes complex. Nat Rev Mol Cell Biol.

[19] Miele L, Miao H, Nickoloff BJ (2006). NOTCH signaling as a novel cancer therapeutic target. Curr Cancer Drug Targets.

[20] Nguyen BC, Lefort K, Mandinova A, Antonini D, Devgan V, Della Gatta G, Koster MI, Zhang Z, Wang J, Tommasi di Vignano A, Kitajewski J, Chiorino G, Roop DR, Missero C, Dotto GP (2006). Cross-regulation between Notch and p63 in keratinocyte commitment to differentiation. Genes Dev.

[21] Wang Z, Zhang Y, Li Y, Banerjee S, Liao J, Sarkar FH (2006). Down-regulation of Notch-1 contributes to cell growth inhibition and apoptosis in pancreatic cancer cells. Mol Cancer Ther.

[22] Das I, Craig C, Funahashi Y, Jung KM, Kim TW, Byers R, Weng AP, Kutok JL, Aster JC, Kitajewski J (2004). Notch oncoproteins depend on gamma-secretase/presenilin activity for processing and function. J Biol Chem.

[23] Kwon C, Cheng P, King IN, Andersen P, Shenje L, Nigam V, Srivastava D (2011). Notch post-translationally regulates beta-catenin protein in stem and progenitor cells. Nat Cell Biol.

[24] Reedijk M, Odorcic S, Zhang H, Chetty R, Tennert C, Dickson BC, Lockwood G, Gallinger S, Egan SE (2008). Activation of Notch signaling in human colon adenocarcinoma. Int J Oncol.

[25] Zhang Y, Li B, Ji ZZ, Zheng PS (2010). Notch1 regulates the growth of human colon cancers. Cancer.

[26] Gao J, Liu J, Fan D, Xu H, Xiong Y, Wang Y, Xu W, Wang Y, Cheng Y, Zheng G (2011). Up-regulated expression of Notch1 and Jagged1 in human colon adenocarcinoma. Pathol Biol (Paris).

[27] Tripathi R, Rath G, Jawanjal P, Sharma S, Singhal P, Bhambhani S, Hussain S, Bharadwaj M (2014). Clinical impact of de-regulated Notch-1 and Notch-3 in the development and progression of HPV-associated different histological subtypes of precancerous and cancerous lesions of human uterine cervix. PLoS One.

[28] Hao L, Rizzo P, Osipo C, Pannuti A, Wyatt D, Cheung LW, Sonenshein G, Osborne BA, Miele L (2010). Notch-1 activates estrogen receptor-alpha-dependent transcription via IKKalpha in breast cancer cells. Oncogene.

[29] Kudo J, Nishiwaki T, Haruki N, Ishiguro H, Shibata Y, Terashita Y, Sugiura H, Shinoda N, Kimura M, Kuwabara Y, Fujii Y (2007). Aberrant nuclear localization of beta-catenin without genetic alterations in beta-catenin or Axin genes in esophageal cancer. World J Surg Oncol.

[30] Mizoguchi K, Ishiguro H, Kimura M, Takahashi H, Sakamoto N, Tanaka T, Takeyama H (2014). Induction of apoptosis by eicosapentaenoic acid in esophageal squamous cell carcinoma. Anticancer Res.

[31] Schroeter EH, Kisslinger JA, Kopan R (1998). Notch-1 signalling requires ligand-induced proteolytic release of intracellular domain. Nature.

[32] Mumm JS, Schroeter EH, Saxena MT, Griesemer A, Tian X, Pan DJ, Ray WJ, Kopan R (2000). A ligand-induced extracellular cleavage regulates gamma-secretase-like proteolytic activation of Notch1. Mol Cell.

[33] Jarriault S, Brou C, Logeat F, Schroeter EH, Kopan R, Israel A (1995). Signalling downstream of activated mammalian Notch. Nature.

[34] Nicolas M, Wolfer A, Raj K, Kummer JA, Mill P, van Noort M, Hui CC, Clevers H, Dotto GP, Radtke F (2003). Notch1 functions as a tumor suppressor in mouse skin. Nat Genet.

[35] Lu Z, Liu H, Xue L, Xu P, Gong T, Hou G (2008). An activated Notch1 signaling pathway inhibits cell proliferation and induces apoptosis in human esophageal squamous cell carcinoma cell line EC9706. Int J Oncol.

[36] Qi R, An H, Yu Y, Zhang M, Liu S, Xu H, Guo Z, Cheng T, Cao X (2003). Notch1 signaling inhibits growth of human hepatocellular carcinoma through induction of cell cycle arrest and apoptosis. Cancer Res.

[37] Balint K, Xiao M, Pinnix CC, Soma A, Veres I, Juhasz I, Brown EJ, Capobianco AJ, Herlyn M, Liu ZJ (2005). Activation of Notch1 signaling is required for beta-catenin-mediated human primary melanoma progression. J Clin Invest.

[38] Reedijk M, Odorcic S, Chang L, Zhang H, Miller N, McCready DR, Lockwood G, Egan SE (2005). High-level coexpression of JAG1 and NOTCH1 is observed in human breast cancer and is associated with poor overall survival. Cancer Res.

[39] Stylianou S, Clarke RB, Brennan K (2006). Aberrant activation of notch signaling in human breast cancer. Cancer Res.

[40] Jundt F, Anagnostopoulos I, Forster R, Mathas S, Stein H, Dorken B (2002). Activated Notch1 signaling promotes tumor cell proliferation and survival in Hodgkin and anaplastic large cell lymphoma. Blood.

[41] Katoh M, Katoh M (2006). Notch ligand, JAG1, is evolutionarily conserved target of canonical WNT signaling pathway in progenitor cells. Int J Mol Med.

[42] Gao F, Huang W, Zhang Y, Tang S, Zheng L, Ma F, Wang Y, Tang H, Li X (2015). Hes1 promotes cell proliferation and migration by activating Bmi-1 and PTEN/Akt/GSK3beta pathway in human colon cancer. Oncotarget.

[43] Bordonaro M, Tewari S, Atamna W, Lazarova DL (2011). The Notch ligand Delta-like 1 integrates inputs from TGFbeta/Activin and Wnt pathways. Exp Cell Res.

[44] Duncan AW, Rattis FM, DiMascio LN, Congdon KL, Pazianos G, Zhao C, Yoon K, Cook JM, Willert K, Gaiano N, Reya T (2005). Integration of Notch and Wnt signaling in hematopoietic stem cell maintenance. Nat Immunol.

[45] Gopalakrishnan N, Saravanakumar M, Madankumar P, Thiyagu M, Devaraj H (2014). Colocalization of beta-catenin with Notch intracellular domain in colon cancer: a possible role of Notch1 signaling in activation of CyclinD1-mediated cell proliferation. Mol Cell Biochem.

[46] Rodilla V, Villanueva A, Obrador-Hevia A, Robert-Moreno A, Fernandez-Majada V, Grilli A, Lopez-Bigas N, Bellora N, Alba MM, Torres F, Dunach M, Sanjuan X, Gonzalez S (2009). Jagged1 is the pathological link between Wnt and Notch pathways in colorectal cancer. Proc Natl Acad Sci U S A.

[47] Duan L, Yao J, Wu X, Fan M (2006). Growth suppression induced by Notch1 activation involves Wnt-beta-catenin down-regulation in human tongue carcinoma cells. Biol Cell.

[48] Ougolkov AV, Yamashita K, Mai M, Minamoto T (2002). Oncogenic beta-catenin and MMP-7 (matrilysin) cosegregate in late-stage clinical colon cancer. Gastroenterology.

[49] Diergaarde B, van Geloof WL, van Muijen GN, Kok FJ, Kampman E (2003). Dietary factors and the occurrence of truncating APC mutations in sporadic colon carcinomas: a Dutch population-based study. Carcinogenesis.

[50] Stahl M, Ge C, Shi S, Pestell RG, Stanley P (2006). Notch1-induced transformation of RKE-1 cells requires up-regulation of cyclin D1. Cancer Res.

[51] Klinakis A, Szabolcs M, Politi K, Kiaris H, Artavanis-Tsakonas S, Efstratiadis A (2006). Myc is a Notch1 transcriptional target and a requisite for Notch1-induced mammary tumorigenesis in mice. Proc Natl Acad Sci U S A.

[52] Weng AP, Millholland JM, Yashiro-Ohtani Y, Arcangeli ML, Lau A, Wai C, Del Bianco C, Rodriguez CG, Sai H, Tobias J, Li Y, Wolfe MS, Shachaf C, Felsher D (2006). c-Myc is an important direct target of Notch1 in T-cell acute lymphoblastic leukemia/lymphoma. Genes Dev.

[53] Palomero T, Lim WK, Odom DT, Sulis ML, Real PJ, Margolin A, Barnes KC, O'Neil J, Neuberg D, Weng AP, Aster JC, Sigaux F, Soulier J (2006). NOTCH1 directly regulates c-MYC and activates a feed-forward-loop transcriptional network promoting leukemic cell growth. Proc Natl Acad Sci U S A.

[54] Lee SH, Jeong EG, Yoo NJ, Lee SH (2007). Mutational analysis of NOTCH1, 2, 3 and 4 genes in common solid cancers and acute leukemias. APMIS.

[55] Weng AP, Ferrando AA, Lee W, Morris JP, Silverman LB, Sanchez-Irizarry C, Blacklow SC, Look AT, Aster JC (2004). Activating mutations of NOTCH1 in human T cell acute lymphoblastic leukemia. Science.

[56] Sharma A, Gadkari RA, Ramakanth SV, Padmanabhan K, Madhumathi DS, Devi L, Appaji L, Aster JC, Rangarajan A, Dighe RR (2015). A novel monoclonal antibody against Notch1 targets Leukemia-associated mutant Notch1 and depletes therapy resistant cancer stem cells in solid tumors. Sci Rep.

